# Type D personality is a risk factor for psychosomatic symptoms and musculoskeletal pain among adolescents: a cross-sectional study of a large population-based cohort of Swedish adolescents

**DOI:** 10.1186/1471-2431-13-11

**Published:** 2013-01-21

**Authors:** Emelie Condén, Jerzy Leppert, Lisa Ekselius, Cecilia Åslund

**Affiliations:** 1 Centre for Clinical Research, Uppsala University, Central Hospital, S-721 89, Västerås, Sweden; 2 Department of Neuroscience, Uppsala University, Uppsala, Sweden

**Keywords:** Adolescents, Musculoskeletal pain, Negative affectivity, Psychosomatic symptoms, Social inhibition, Type D personality

## Abstract

**Background:**

Type D personality, or the “distressed personality”, is a psychosocial factor associated with negative health outcomes, although its impact in younger populations is unclear. The purpose of this study was to investigate the prevalence of Type D personality and the associations between Type D personality and psychosomatic symptoms and musculoskeletal pain among adolescences.

**Methods:**

A population-based, self-reported cross-sectional study conducted in Västmanland, Sweden with a cohort of 5012 students in the age between 15–18 years old. The participants completed the anonymous questionnaire Survey of Adolescent Life in Västmanland 2008 during class hour. Psychosomatic symptoms and musculoskeletal pain were measured through index measuring the presence of symptoms and how common they were. DS14 and its two component subscales of negative affectivity (NA) and social inhibition (SI) were measured as well.

**Results:**

There was a difference depending on sex, where 10.4% among boys and 14.6% among girls (p = < 0.001) were defined as Type D personality. Boys and girls with a Type D personality had an approximately 2-fold increased odds of musculoskeletal pain and a 5-fold increased odds of psychosomatic symptoms. The subscale NA explained most of the relationship between Type D personality and psychosomatic symptoms and musculoskeletal pain. No interaction effect of NA and SI was found.

**Conclusions:**

There was a strong association between Type D personality and both psychosomatic symptoms and musculoskeletal pain where adolescent with a type D personality reported more symptoms. The present study contributes to the mapping of the influence of Type D on psychosomatic symptoms and musculoskeletal pain among adolescents.

## Background

The high prevalence of musculoskeletal and psychosomatic symptoms among adolescents in the western world is a problem involving significant costs for both individuals and societies [1,2]. Musculoskeletal pain and psychosomatic symptoms that appear during adolescence often persist into adulthood and may partly be explained by psychosocial and lifestyle factors [3-5]. Pain among adolescents has been identified as an important public health problem. Roth-Isigkeit found that 83% of children and adolescents had experienced pain during the preceding three months, with headache, abdominal, limb and back pain being the most prevalent types. Pain caused the respondents of the Roth-Isigkeit study several restrictions on their lives [6]. A growing body of literature suggests that Type D personality is a psychosocial factor associated with negative health outcomes. The Type D personality, or the “distressed personality”, is characterised by two global personality traits: negative affectivity (NA) and social inhibition (SI). For clinical use, this personality type has been associated with a variety of emotional and social difficulties and increased morbidity and mortality in patients with established cardiovascular disease [7-11]. Merlijn showed that adolescents with chronic functional somatic complaints scored higher on neuroticism (a scale with many resemblances to the Type D personality) compared with their peers devoid of any chronic complaints [12]. This implies that personality characteristics may be associated with somatic complaints even in younger age groups. There is a strong association between self-reported somatic complaints and poorer psychological well-being in adolescents [13,14]. Given the relationship between psychological well-being and self-perceived physical health, it seems possible that personality traits are associated with somatic complaints. Poor health that manifests itself at a young age may have a significant impact on an individual’s clinical situation in adulthood. Studies investigating the association between personality and physical and mental health in adolescents are, however, scarce. The aim of the present study was, therefore, to investigate the prevalence and associations between Type D personality, psychosomatic symptoms, and musculoskeletal pain in a large, representative, adolescent population.

## Methods

**Figure 1:**
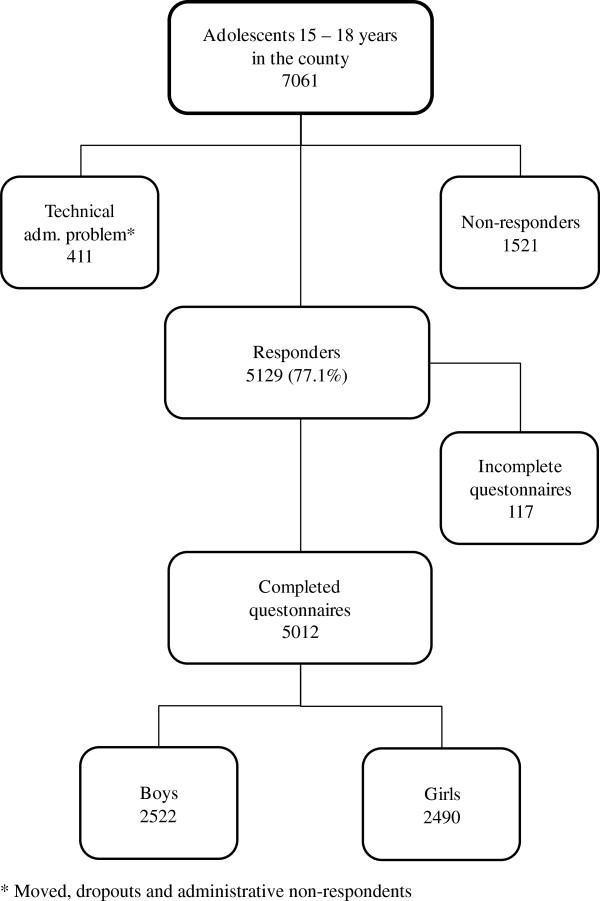
Flow chart of the study population.

The study was performed among secondary school students in the county of Västmanland, Sweden, during 2008. All students in the ninth grade of elementary school (15–16 years old) and the second year of secondary school (17–18 years old) were asked to complete a self-report questionnaire during school time. The questionnaire was part of the Survey of Adolescent Life in Vestmanland 2008 (SALVe 2008), a survey distributed biannually by the County Council of Västmanland in order to monitor the psychosocial health of the adolescent population of the county. Västmanland is a middle-sized county situated about 100 kilometres west of Stockholm and, due to its mix of rural and urbanized areas, can be considered a representative example of Sweden as a whole. The survey included questions about demographic background, psychosomatic and musculoskeletal symptoms and Type D personality. Further information about the sample is given in Figure 1. The study was approved by the regional human ethical review board of Uppsala University and followed the Swedish guidelines for social sciences and humanities studies according to the Declaration of Helsinki. Participation was anonymous and voluntary. 

### Measures

*The demographic data* were measured by several different variables: Sex (boy: 1, girl: 2). Parental employment status was divided into two groups: 1: whether both of the parents were working, 2: if one or both of the parents were unemployed. Ethnicity was classified into two groups: 1: both parents were born in Sweden or Scandinavia, 2: one or both of the parents were born outside of Scandinavia. Housing conditions: 1: the participant was living in a single-family house, 2: in a multi-family house. Socioeconomic status was reported on a 7-point Likert scale with the following question: Imagine society as being like a ladder. At the bottom are those with the least money (1), and at the top are those with the most (7). If you think about how wealthy your own family is compared with the rest of society, where would you place them on this scale? [15].

*Body Mass Index* was defined for each participant. The participant reported his or her weight and length, and the BMI index was calculated. The BMI index expresses relative weight and is defined as an individual's weight divided by height squared kg/m2. The index was divided into three groups: < 18 underweight, 18.01-24.99 normal weight, >25 overweight/obesity.

#### Exercise

The participants were divided by exercise habits: at least 30 minutes of exercise at least once a week (1), and exercise less than once a week (2).

#### Type D personality

Personality was measured using the DS14 [16]. It consists of two 7-item subscales and measures negative affectivity (NA), social inhibition (SI), and Type D personality. The respondents rated their personality on a 5-point Likert scale ranging from 0: false,1: rather false, 2: neutral, 3: rather true and 4: true, with a maximum score of 28 on each scale. A cut-off of 10 or more on both scales is used to classify a respondent as a Type D personality. Denollet based the cut-off on the median split in representative samples. Clinical evidence for this cut-off based classification was obtained in longitudinal clinical studies and empirical evidence from latent class cluster analysis [17]. The DS14 is a valid and reliable scale with a Cronbach’s α of 0.88/0.86 and a 3-month test-retest reliability of (*r*) = 0.72/0.82 for the NA and SI subscales in an adult population [16]. In the present study, Cronbach’s α was 0.86/0.79. It has been suggested that type D personality may be better represented as a dimensional construct [18], and should be analyzed as the interaction between negative affectivity and social inhibition [19,20]. We therefore created a variable with the total raw score based on the NA × SI interaction term which are used in the descriptive tables as a complement. We also performed Z-score transformations of the NA and SI subscales to test for interaction effects in the logistic regressions.

#### Psychosomatic symptoms

The participants were asked how often they suffered from 1: headache, 2: stomach ache, 3: feelings of nervousness, 4: feelings of irritation, 5: sleep problems. The participants rated their alternatives from: never: 0, seldom: 1, sometimes: 2, often: 3, always: 4. A summation index was created with a range of 0–20 points. The use of this measurement has previously been reported in Åslund et al. [21]. The internal consistency (Chronbach’s alpha) of the psychosomatic symptoms questions was 0.75. The index was then divided by standard deviations, where +1 SD was the cut-off for many and – 1 SD the cut-off for few psychosomatic symptoms. The intermediate group (medium symptoms) and the group with few psychosomatic symptoms were then merged into one group labelled as few-medium psychosomatic symptoms.

#### Musculoskeletal pain

The participants were asked how often they suffered from 1: pain in the shoulders/neck, 2: pain in the back/hips, 3: pain in the hands/knees/legs and feet. The participants rated their alternatives from: never: 0, seldom: 1, sometimes: 2, often: 3, always: 4. A summation index was created with a range of 0–12 points. The use of this measurement has previously been reported in Åslund et al. [21]. The internal consistency (Chronbach’s alpha) of the psychosomatic symptoms questions was 0.69. The index was then divided by standard deviations, where +1 SD was the cut-off for many and – 1 SD the cut-off for few musculoskeletal pain symptoms. The intermediate group (medium symptoms) and the group with few musculoskeletal pain symptoms were then merged into one group labelled as few-medium musculoskeletal pain symptoms.

### Statistical analyses

Sex differences in the demographic factors were analysed with the Mann–Whitney and x^2^ test. Correlations between the factors of the model were performed with Spearman’s Rho. To investigate the associations between Type D personality, psychosomatic symptoms, and musculoskeletal pain binary logistic regression was used as well as linear regression. The binary logistic regression resulted in an odds ratio to declare a way of comparing whether the probability of a certain event was the same for two groups. We moreover included Z-score transformations of the NA and SI scales and tested for interaction effects. The interaction term in the binary logistic regressions were based on the Z-transformed NA (Z(NA)) × Z-transformed SI (Z(SI)) scores. All statistical analyses were performed using SPSS 18.0.

## Results

Demographic data and BMI are presented in Table 1. There were no significant differences between boys and girls except for boys reporting higher socioeconomic status (boys *M* = 4.39, girls *M* = 4.26, *Z* = − 4.207, *p* <0.001), and in the categories BMI < 18 and > 25, girls were more frequent in the lower categories. Boys had a mean BMI of 22.2 and girls 21.0 (*Z* = −12.78, *p* <0.001). A total of 625 (12.5%) of the study population were defined as Type D personality. There was a difference depending on sex, where 10.4% of boys and 14.6% of girls (*p* = < 0.001) were defined as a Type D personality. Regarding the two subscales of the DS14, 457 (18.3%) of the boys and 870 (35.0%) of the girls (x^2^ = 179.19 *p* = < 0.001) had a score above the cut-off value of 10 points on the NA subscale, and 537 (21.5%) of the boys and 591 (23.8%) of the girls had a score above the cut-off value of 10 points on the SI subscale (x^2^ = 3.77 *p* = 0.052). Concerning psychosomatic symptoms, 248 (9.9%) of the boys and 756 (30.4%) of the girls reported having many symptoms (*Z* = −26.813, *p* <0.001). Regarding musculoskeletal pain, 228 (9.1%) of the boys and 512 (20.6%) of the girls reported having many symptoms (*Z* = −15.220, *p* <0.001). Means, medians, SDs and p-values are reported in Table 2. 

**Table 1:** **Demographic characteristics and BMI of the study population**^**a**^

	**Boys**		**Girls**			
	***n***	**%**	***n***	**%**	***Χ***^***2***^	***p***
*Ethnicity*						
Scandinavian ethnicity	2088	83.9	2067	83.6	0.106	0.745
Non-Scandinavian ethnicity	401	16.1	405	16.4	0.020	0.888
*Parental employment status*						
Both parents working	1982	79.8	1934	78.0	0.588	0.443
At least one parent unemployed	501	20.2	544	22.0	1.769	0.183
*Housing conditions*						
Living in an apartment	659	26.3	672	27.1	0.127	0.722
Living in a villa etc.	1851	73.7	1809	72.9	0.482	0.488
*Exercise habits*						
Exercises at least once a week	1904	78.4	1872	77.4	0.271	0.603
Exercises less than once a week	524	21.6	581	23.7	2.940	0.086
*BMI*						
BMI < 18	154	6.4	287	12.4	40.111	<0.001
BMI 18–24.99	1845	77.1	1836	79.3	0.022	0.882
BMI >25	394	16.5	191	8.3	70.443	<0.001

^a^Split on boys and girls, with p-values of sex differences.

**Table 2:** Correlations between the NA × SI interaction variable, social inhibition (SI), negative affectivity (NA), psychosomatic symptoms, and musculoskeletal pain

	**NA** **×** **SI**	**SI**	**NA**	**Psychosomatic symptoms**	**Musculoskeletal pain**
NA × SI ^c^	-	0.845*^a^	0.893*	0.467*	0.289*
SI ^d^	0.841*^b^	-	0.532*	0.256*	0.174*
NA ^e^	0.864*	0.475*	-	0.541*	0.316*
Psychosomatic symptoms	0.513*	0.253*	0.623*	-	0.436*
Musculoskeletal pain	0.294*	0.140*	0.360*	0.425*	-

Boys are presented above the diagonal, and girls below the diagonal.

*Significant level at p = <0.01.

^a^Boys are presented above the diagonal.

^b^Girls are presented below the diagonal.

^c^Type D personality total raw score based on the NA × SI interaction term.

^d^Social inhibition raw score.

^e^Negative affectivity raw score.

**Table 3:** **Main outcome measurements for the indices of the study**^**a**^

	**Boys**			**Girls**			
	**Mean**	**Median**	**SD**	**Mean**	**Median**	**SD**	**P**
Psychosomatic symptoms	6.97	7.00	3.54	9.73	10.00	3.41	< 0.001
Musculoskeletal pain	3.12	3.00	2.51	4.27	4.00	2.71	< 0.001
Type D personality, NA × SI ^b^	245.54	210.00	150.54	296.37	264.00	168.74	< 0.001
Type D personality, SI ^c^	15.87	16.00	4.74	16.20	16.00	5.04	0.089
Type D personality, NA ^d^	14.57	14.00	5.35	17.41	17.00	5.72	< 0.001

^a^Means, medians, SDs, 95% CIs, and quartiles, separated by boys and girls with p-values for sex differences.

^b^Type D personality total raw score based on the NA × SI interaction term.

^c^Social inhibition raw score.

^d^Negative affectivity raw score.

As shown in Table 3, there was a moderate association between Type D personality and psychosomatic symptoms, and a somewhat weaker correlation with musculoskeletal pain among both boys and girls. The NA subscale accounted for the strongest correlation regarding both psychosomatic symptoms and musculoskeletal pain. When analysing boys and girls together, there were correlations between the non-independent confounding factors (living conditions, parental employment status, ethnicity, socioeconomic status, BMI, and exercise habits) and Type D personality, psychosomatic symptoms and musculoskeletal pain varying between *r =* 0.034, *p* = 0.018 (Type D personality and living conditions), to *r = −*0.195*, p* <0.001 (Type D personality and SES). Moreover, there was a weak relation between parental unemployment and socioeconomic status (*r* = −0.217, *p* = <0.001).

In a logistic regression, Type D personality was found to be associated with both psychosomatic symptoms and musculoskeletal pain (Table 4). Both boys and girls with a Type D personality had an approximately 2-fold increased risk of musculoskeletal pain and a 5-fold increased risk of psychosomatic symptoms. 

**Table 4:** Logistic regression of having many musculoskeletal symptoms, or many psychosomatic symptoms in relation to Type D personality, odds ratio (OR), 95% CI, p-values, unadjusted model, and model adjusted for confounding factors

	**Boys**						**Girls**					
	**Unadjusted model**			**Adjusted model**^**a**^			**Unadjusted model**			**Adjusted model**^**a**^		
	**OR**^**b**^	***95% CI***	***p***	**OR**^**b**^	***95% CI***	***p***	**OR**^**b**^	***95% CI***	***p***	**OR**^**b**^	***95% CI***	***p***
**Model 1**												
*Many musculoskeletal symptoms*												
Type D personality	2.42	1.70-3.44	<0.001	2.39	1.62-3.52	<0.001	2.46	1.93-3.13	<0.001	2.38	1.82-3.11	<0.001
	*R*^*2*^ = 1.8%			*R*^*2*^ = 3.4%			*R*^*2*^ = 3.1%			*R*^*2*^ = 4.4%		
*Many psychosomatic symptoms*												
Type D personality	5.74	4.23-7.80	<0.001	5.39	3.83-7.57	<0.001	5.43	4.29-6.88	<0.001	5.54	4.26-7.21	<0.001
	*R*^*2*^ = 9.0%			*R*^*2*^ = 11.7%			*R*^*2*^ = 11.4%			*R*^*2*^ = 14.4%		
**Model 2**												
*Many musculoskeletal symptoms*												
Z(SI) ^c^	0.92	0.77-1.09	0.327	0.89	0.73-1.08	0.231	0.79	0.69-0.89	<0.001	0.80	0.69-0.91	0.001
Z(NA) ^d^	2.04	1.75-2.39	<0.001	2.08	1.76-2.47	<0.001	2.25	1.99-2.54	<0.001	2.19	1.92-2.50	<0.001
Z(SI) × Z(NA)	0.95	0.86-1.05	0.298	0.95	0.85-1.07	0.414	1.07	0.98-1.17	0.133	1.08	0.99-1.19	0.093
	*R*^*2*^ = 8.4%			*R*^*2*^ = 9.7%			*R*^*2*^ = 12.9%			*R*^*2*^ = 13.3%		
*Many psychosomatic symptoms*												
Z(SI) ^c^	0.97	0.77-1.19	0.797	0.98	0.79-1.23	0.887	0.83	0.73-0.95	0.006	0.89	0.77-1.01	0.068
Z(NA) ^d^	3.58	3.00-4.27	<0.001	3.44	2.85-4.16	<0.001	4.56	3.95-5.25	<0.001	4.40	3.78-5.12	<0.001
Z(SI) × Z(NA)	0.89	0.80-1.00	0.058	0.91	0.80-1.04	0.157	1.05	0.93-1.19	0.390	1.05	0.92-1.19	0.487
	*R*^*2*^ = 25.2%			*R*^*2*^ = 26.5%			*R*^*2*^ = 36.7%			*R*^*2*^ = 37.4%		

^a^ Adjusted for living conditions, parental employment status, ethnicity, socioeconomic status, BMI, and exercise habits.

^b^ Nagelkerke *R*^*2*^ is given for the analysis of each symptom category, separated by sex.

^c^ Social inhibition, Z-score transformation.

^d^ Negative affectivity, Z-score transformation.

The unadjusted model did not differ markedly from a model adjusted for possible non-independent confounding factors such as living conditions, parental unemployment, ethnicity, socioeconomic status, BMI, and exercise habits. However the Nagelkerke *r*^*2*^ increased somewhat in the adjusted models, being stronger for psychosomatic symptoms (11–14%) compared with musculoskeletal pain (3.4-4.4%). When analysing the Z-score transformations of NA and SI in a logistic regression interaction model, the NA scale was associated with an increased risk of having many musculoskeletal symptoms (Table 4, Model 2). The SI scale was however associated with a decreased risk of musculoskeletal symptoms among girls. No significant interaction effects were found neither among boys or girls in relation to musculoskeletal symptoms. A similar pattern was found regarding psychosomatic symptoms, were NA was associated with a four-fold increased risk among boys and girls, but no significant effect was found for SI or the interaction between NA and SI in the adjusted models. We also performed a linear regression which confirmed these results with no significant effect of SI in relation to musculoskeletal pain, and only a weak association between SI and psychosomatic symptoms (data not shown).

## Discussion

The aim of this study was to investigate the prevalence of Type D personality and its associations with psychosomatic symptoms and musculoskeletal pain among adolescents. Among adolescents in the age between 15–18 years 12.5% were classified as type D personality. Moreover, there was a strong association between this personality type and both psychosomatic symptoms and musculoskeletal pain, with adolescents with a Type D personality reporting more symptoms. When the two subscales of Type D personality were analysed separately, it was found that the NA subscale accounted for the major part of the association. The findings of associations between Type D personality, psychosomatic symptoms, and musculoskeletal pain confirm Jellesma’s assumption that Type D personality characteristics are positively associated with self-reported somatic complaints among adolescents [22]. The findings also resemble those of Mols and Denollet, where Type D personality was associated with an increased number of health complaints and heightened perception of negative emotions such as depression [23]. Several studies have shown that Type D characteristics have a negative impact on both mental and physical health status such as somatic complaints and social support [23,24]. Type D personality has been suggested as a vulnerability factor for general psychological distress that affects both mental and physical health status among adults [7]. It has also been associated with poor self-management of disease [25]. Adolescents with this personality type seem to experience longer and more intense psychophysiological reactions than adolescents without it. One possible explanation for the relation between Type D personality and psychophysiological reaction may be how the body copes with stress through physiological functions, and the allostatic load. Chronic exposure to stress gives a constant stimulation of the HPA axis which results in high levels of cortisol [26,27]. Denollet et al. suggest that personality might be linked to health outcome either directly through psychophysiological mechanisms, such as those above, or indirectly through poor health behaviours or psychological factors, such as a lack of social support that may arise from the personal behaviour Type D personality results in [10]. The behavioural strategies of withdrawal and avoidance independent from their motives are probably related to a smaller number of peer relationships and less social support [28]. It has been reported that 13-24% of individuals in a healthy adult population can be classified as a Type D personality [16,29,30]. A previous study on young people has shown a similar pattern [22]. The present study showed a lower prevalence of Type D personality among adolescents. Although both boys and girls scored relatively high on both subscales (i.e. 35% of the girls had a score above cut-off on the NA subscale) relatively few met the cut-off for Type D personality. Jellesma suggested that Denollet’s criteria may be too sensitive for adolescents with scores around the median, because the characteristics of Type D personality become more stable during adolescence and manifest themselves around the age of 20 [22]. As described earlier, there was a sex difference in the prevalence of Type D personality, with a preponderance of girls. Few previous studies regarding this personality type have highlighted differences between the sexes and least of all, regarding adolescents. Kupper et al. found that the prevalence of Type D personality was higher among women [31]. Moreover, females had significantly higher NA scores than males but males and females did not differ in their mean SI scores. Age was not a significant covariate for either NA or SI [31]. As the subscales of Type D personality differed in strength of correlations in relation to the outcome variables, we chose to further investigate the importance of each subscale. In the present study, the association between Type D personality, psychosomatic symptoms, and musculoskeletal pain was primarily explained, among both sexes, by the NA subscale. The association between the SI subscale and the studied symptoms was weak. NA denotes a stable tendency to experience negative emotions [32]. Individuals with a high level of NA often experience a feeling of dysphoria, anxiety, irritability and apprehension. SI denotes a stable tendency to inhibit the expression of emotions and behaviours in social interaction [33]. Individuals with a high level of SI feel inhibited, insecure, and tense when interacting socially with others [34]. Furthermore, individuals with a high level of both NA and SI seem to scan their surroundings for signs of imminent trouble [35] and to avoid negative reactions [36]. Trapped in negative emotions, an individual with a Type D personality experiences high stress levels, and social inhibition prevents the individual from obtaining help necessary to relieve the stress. Moreover, it has been suggested that Type D personality should be analyzed as the interaction between negative affectivity and social inhibition [19,20], and we therefore included interaction analyses in the logistic regressions. However, in the present study no significant interaction effects of NA x SI were found in relation to musculoskeletal pain or psychosomatic symptoms. One possible explanation may be the strong influence of negative affect in relation to the outcome variables. A high level of NA has been shown to contribute to depression among adolescent girls [37]. Previous studies have also shown that the NA subscale is the one most associated to negative clinical outcome [20,38]. However, although no significant interaction effects were found, the explained variance according to Nagelkerke’s *R*^*2*^ increased dramatically in the interaction models compared to the analyses of the dichotomized Type D personality variable. This might be explained by the finding that SI was associated with a decreased risk for musculoskeletal pain and psychosomatic symptoms, whereas NA was associated with an increased risk, especially among girls.

Girls reported significantly more musculoskeletal pain and psychosomatic symptoms than boys, which are in accordance with previous studies [39,40]. Pain triggers have been suggested to differ between the sexes [6], with boys often stating that their pain was triggered by physical exertion, while with girls, the trigger was often a common cold or internal factors such as anger disputes, family conditions or sadness [6]. Furthermore, previous studies have shown patterns where women seem to show lower pain thresholds, higher pain ratings, and a lower tolerance for pain [41]. Barnett showed that Type D personality and its two subscales demonstrate strong internal consistency among chronic pain patients [42].

The results of this study need to be interpreted in the light of several limitations. Firstly, the study relies exclusively on self-reports by adolescents on questionnaires assessing Type D personality, psychosomatic symptoms, and musculoskeletal pain. When relying upon self-reported material, there is always a risk of information bias, such as false-positive or false-negative reports, from the participants. Although the questionnaire was well-suited for the aims of the study, the answers given may, for example, be inconsistent with the perceptions of the adolescents’ parents and teachers. Moreover, questionnaire studies on school populations may miss those students with the most psychosomatic symptoms or musculoskeletal pain due to their absence from school on the day of the questionnaire. Given their inhibited nature and passive coping style, it is possible that Type D individuals may have been less likely to participate in the questionnaire [43]. Secondly, no consideration has been given to diseases or other states that can affect psychosomatic symptoms or musculoskeletal pain. Moreover, we did not use a diagnostic instrument to measure psychosomatic symptoms and musculoskeletal pain, but rather a report of the subjective feelings of the symptoms and pain. No information was available on the use, admittedly rare among this population, of psychotropic medications or participation in rehabilitation programs which can affect the experiences of musculoskeletal pain and psychosomatic symptoms. Thirdly there is always a risk of other explanatory factors not included in our models. For example, according to Deubner menarche is associated with physical symptoms such as menstrual cramps, headache and migraine [44]. An important consideration in the interpretation of our results is that Fishbain et al. showed that the personality characteristics of patients with pain changed with the pain treatment they received [45]. One alternative explanation needed to be considered is whether adolescents with Type D personality report more symptoms than others. Often there are no real differences between individuals with high degrees of neuroticism and those with low levels when objective health measurements are made. This demonstrates the powerful force of “self-perceived health” (an individual’s perception of their own health), which has been linked with both health status and health consequences [46]. Additionally, when using a cross-sectional method, it is always difficult to give any causal relationship.

This study also has several strengths. Firstly, it is a population-based design and had a particularly satisfactory participation rate. The results of this study reflect the associations between Type D personality, psychosomatic symptoms, and musculoskeletal pain among adolescents in the present cohort, but the high participation rate also provides an excellent opportunity to generalize the results to other adolescent populations as well. Additionally, the sample size and rate of participation gives a high statistical power. As the participants included all students in ninth grade of elementary school (15–16 year olds) and the second year of secondary school (17–18 year olds), the study constitutes a reliable community sample of this age group in a middle-sized urbanized region of Sweden.

Further longitudinal studies are needed to clarify whether Type D personality and its traits remain stable over time and after life-changing events. Moreover, the influence, through disease promoting mechanisms, of Type D personality on health status in later life needs to be investigated.

## Conclusions

In the present study, more than every tenth adolescent from the general population was classified as a Type D personality. This personality type, or in particular NA, may be an important factor in increasing the risk of suffering from musculoskeletal pain and, above all, psychosomatic symptoms among adolescents. It may be beneficial, through targeted behavioural interventions, to identify these adolescents and to decrease not only the stress that they may suffer from, but also lower their perception of negative emotions.

## Abbreviations

NA: Negative affectivity; SI: Social inhibition.

## Competing interests

All authors have been provided a copy of this submission. The paper reports original research that has not been published before in any form. The paper is not under concurrent consideration for publication elsewhere in whole or in part in any language.

All applicable subject protection guidelines and regulations were followed in the conduct of the research. All authors declare no conflicts of interest and no financial interests in the publishing of the manuscript.

## Authors’ contributions

EC, CÅ, JL and LE were responsible for the concept and design of the study. CÅ and JL contributed to the acquisition of the data and CÅ performed the questionnaire management. EC and CÅ performed the data analysis and interpretation of the findings, and EC wrote the manuscript under the supervision of CÅ. JL and LE provided critical revision of the manuscript, and all authors reviewed and approved the final version for publication. CÅ had full access to all the data in the study and takes responsibility for the integrity of the data and the accuracy of the data analysis.

## Pre-publication history

The pre-publication history for this paper can be accessed here:

http://www.biomedcentral.com/1471-2431/13/11/prepub
